# Speckle-tracking echocardiography for predicting improvement of myocardial contractile function after revascularization: a meta-analysis of prospective trials

**DOI:** 10.1007/s10554-022-02753-2

**Published:** 2022-11-11

**Authors:** Haitham Ballo, Fatma Doghman, Juha Hartikainen, Antti Saraste, Juhani Knuuti

**Affiliations:** 1 Turku PET Centre, University of Turku and Turku University Hospital, Turku, Finland; 2 Heart Center, Turku University Hospital, Turku, Finland; 3 Department of Pulmonary Diseases, Turku University Hospital, Turku, Finland; 4 Heart Center, Kuopio University Hospital and University of Eastern Finland, Kuopio, Finland

**Keywords:** 2D-Speckle tracking echocardiography, Revascularization, Myocardial viability

## Abstract

Some studies have indicated that the use of 2D-Speckle tracking echocardiography (2DSTE) aids in predicting recovery of myocardial contractile function after revascularization in patients with chronic ischemic left ventricular (LV) dysfunction or acute myocardial infarction (MI). The purpose of this meta-analysis was to evaluate the diagnostic accuracy of 2DSTE strain in the detection of myocardial viability at rest and during low-dose dobutamine (LDD) stress. A systematic review for all prospective trials using 2DSTE to assess myocardial viability until January 2019 was done. Using a standard approach of meta-analysis for diagnostic tests. Overall, nine studies including 525 patients with either chronic ischemic heart disease or acute MI fulfilled the inclusion criteria. Seven studies used longitudinal strain (LS) at rest, nine studies used circumferential strain (CS) at rest, four studies used LS during LDD stress, and four studies used CS during LDD stress. LS and CS during LDD stress showed equally high sensitivity (81.5% and 81.5% respectively) and specificity (81.3% and 81.4% respectively) for detecting reversible dysfunction. At rest, LS and CS showed equally lower sensitivity (67.1%, *p* < 0.0001 vs. LDD stress and 68.7%, *p* < 0.0001, vs. LDD stress, respectively) and specificity (64%, *p* < 0.0001 vs. LDD stress and 65.7%, *p* = 0.0008 vs. LDD stress, respectively) as compared with LDD stress. LS and CS by 2DSTE during LDD stress accurately identify reversible ischemic myocardial dysfunction in patients with chronic ischemic LV dysfunction or after MI. The use of LDD stress can be recommended over resting strain measures in this setting.

**Supplementary Information:**

The online version contains supplementary material available at 10.1007/s10554-022-02753-2.

## Introduction

Ischemic myocardium that is dysfunctional, but viable has potential for recovery of contractile function after revascularization [1]. The assessment of myocardial viability in patients with ischemic left ventricular (LV) dysfunction has prognostic implications [2] and informs selection of the therapeutic strategy. Current guidelines recommend to consider myocardial revascularization in patients with chronic ischemic heart failure with EF ≤ 35% in the presence of viable myocardium [3–5].

Several cardiac imaging modalities have been used to detect myocardial viability [1], including dobutamine stress echocardiography. Assessment of myocardial deformation by 2D-speckle-tracking echocardiography (2DSTE) enables objective measurement of systolic function at rest and during dobutamine stress [6–8]. Recent studies have indicated that 2DSTE measures of myocardial strain are useful for the detection of reversible ischemic LV dysfunction [9–11]. So far, 2DSTE technique has not been included in meta-analyses assessing the performance of different tests in the detection of viability [1]. Furthermore, individual studies on 2DSTE have included relatively small patient populations and provided limited information on factors influencing the performance of 2DSTE, such as the relative performance of strain at rest or low-dose dobutamine (LDD) stress [10, 12–14].

In order to clarify the value of 2DSTE in the assessment of viability, we performed a meta-analysis to evaluate the accuracy of 2DSTE with or without a LDD stress for predicting recovery of regional myocardial contractile function after revascularization in chronic ischemic LV dysfunction or after an acute MI.

## Methods

### Search strategy

We did a systematic search for original studies published until January 2019 using PubMed, Cochrane Central Register of Clinical Trials, and Embase using terms: (speckle tracking echocardiography OR myocardial deformation OR strain) AND (myocardial infarction OR viability OR viability test OR viable myocardium) AND (coronary revascularization). The search results were limited to the English language and to studies performed in humans. Trials in the abstract form without a manuscript published as well as case reports, review articles, editorials, and expert opinions were excluded.

### Selection criteria

Trials included in the analysis had to meet the following criteria: (i) study approved by institutional review boards (IRBs); (ii) prospective study utilizing regional longitudinal or circumferential strain to predict the recovery of LV function after coronary revascularization (i.e., PCI or CABG); (iii) wall motion analysis (WMA) by echocardiography was used as a reference standard to identify myocardial segments with functional recovery after the revascularization (defining functional recovery based on the improvement of wall motion score by at least 1 grade); (iv) allowed for calculation of sensitivity, specificity, positive likelihood ratio (LR +) and negative likelihood ratio (LR-) based on segmental analysis. The LR + and LR- were calculated as sensitivity/ (1–specificity) and (1 -sensitivity) /specificity.

### Data extraction

Two investigators (H.B and F.D) extracted the data independently using a standardized protocol and reporting form. Year of publication, echocardiographic characteristics of each trial, baseline demographics, the interval between revascularization and follow-up, as well as the number of viable and nonviable segments predicted in each exam were extracted. The absolute numbers of true positives, false positives, true negatives and false negatives were extracted from the data. Studies were grouped according to the 2DSTE tests, which included longitudinal strain (LS) at rest, LS during LDD stress, circumferential strain (CS) at rest, circumferential strain during LDD stress, radial strain (RS) at rest and RS during LDD stress. If a study compared more than one modality to the reference standard, each test was evaluated separately.

### Quality assessment

To assess the quality and reporting of studies, we evaluated 14 items that were considered relevant to the review topic, based on the modified Quality Assessment of Studies of Diagnostic Accuracy Included in Systematic Reviews–two criteria (QUADAS-2) [15]. Two reviewers (H.B. and F.D.) independently assessed patient spectrum, reference standard, disease progression bias, verification bias, review bias, clinical review bias, incorporation bias, test execution, study withdrawals, and indeterminate results.

### Statistical analysis

We followed the guidelines provided by the Cochrane Collaboration for systematic reviews of diagnostic test accuracy [16]. The statistical analysis for such reviews focuses on two statistical measures of diagnostic accuracy of the test sensitivity and specificity. We decided a priori to analyse the data using the bivariate random-effects model because of the anticipated heterogeneous nature of the included studies. Bivariate random-effects models were constructed to combine individual study-level data on the sensitivities and specificities across studies. This model takes into account the correlation between sensitivity and specificity, assesses heterogeneity across studies in sensitivity and specificity, and allows to compare sensitivity and specificity between the methods [17]. The bivariate model used parameters to extract summary points for sensitivity and specificity with 95% confidence intervals (CIs). We performed the summary receiver operator characteristic (ROC) graph by the relationship between sensitivity and specificity and then derived summary estimates of the LR + and LR- with their CIs from the model estimates. Since sensitivity and specificity in bivariate model are correlated, univariate measures of heterogeneity, such as *I*^2^, are typically not recommended to report heterogeneity in diagnostic test accuracy reviews [16]. Thus, we assessed between-study heterogeneity visually by plotting sensitivity and specificity in the forest plots and ROC curves. We also conducted meta-regression regarding mean age, male involved percentage, mean LVEF, LDD stress in the study, follow-up time after the revascularization, and time interval between MI onset and 2DSTE analysis. We wanted to evaluate the influence of these factors on the diagnostic power of LS and CS. As a part of sensitivity analysis [16], we made a direct comparison to ensure that an unbiased comparison was restricted to studies that made head-to-head comparisons (i.e., applying two of the tests to the same patients). We conducted a further sensitivity analysis by omitting outlier studies. We used the ad hoc methods (sensitivity and specificity plots) and interquartile range (1.5 IQR) rule to detect outlying studies. Furthermore, we derived strain values to compare segments showing functional recovery at follow-up with those that did not in each test. The values were determined by the authors of each individual study and formed the basis on which the 2DSTE parameters before revascularization for all dyskinetic segments related to functional status at follow-up. Furthermore, for the semiquantitative comparison between segments with and without functional recovery, the pooled mean strain values, and the mean differences (MD) were estimated with 95% CIs. We did the statistical analyses by R version 3.2.3 (R Foundation for Statistical Computing, Vienna, Austria) using the mada package (https://Cran.R-project.org/package=mada). We obtained a summary ROC graph through the Review Manager (RevMan; Cochrane Collaboration; release 5.3) using the parameters gained from R. We performed meta-regression, and the pooled mean strain values and MD analysis using Open Meta-Analyst software (Version 10.10) by the Center for Evidence-Based Medicine (CEBM) of Brown University [18]. All tests were two-sided, and statistical significance was defined as a *P*-value < 0.05. Publication bias was not assessed due to the number of studies being less than ten in each test group, according to Higgins and Green[19].

## Results

### Study selection

**Fig. 1:**
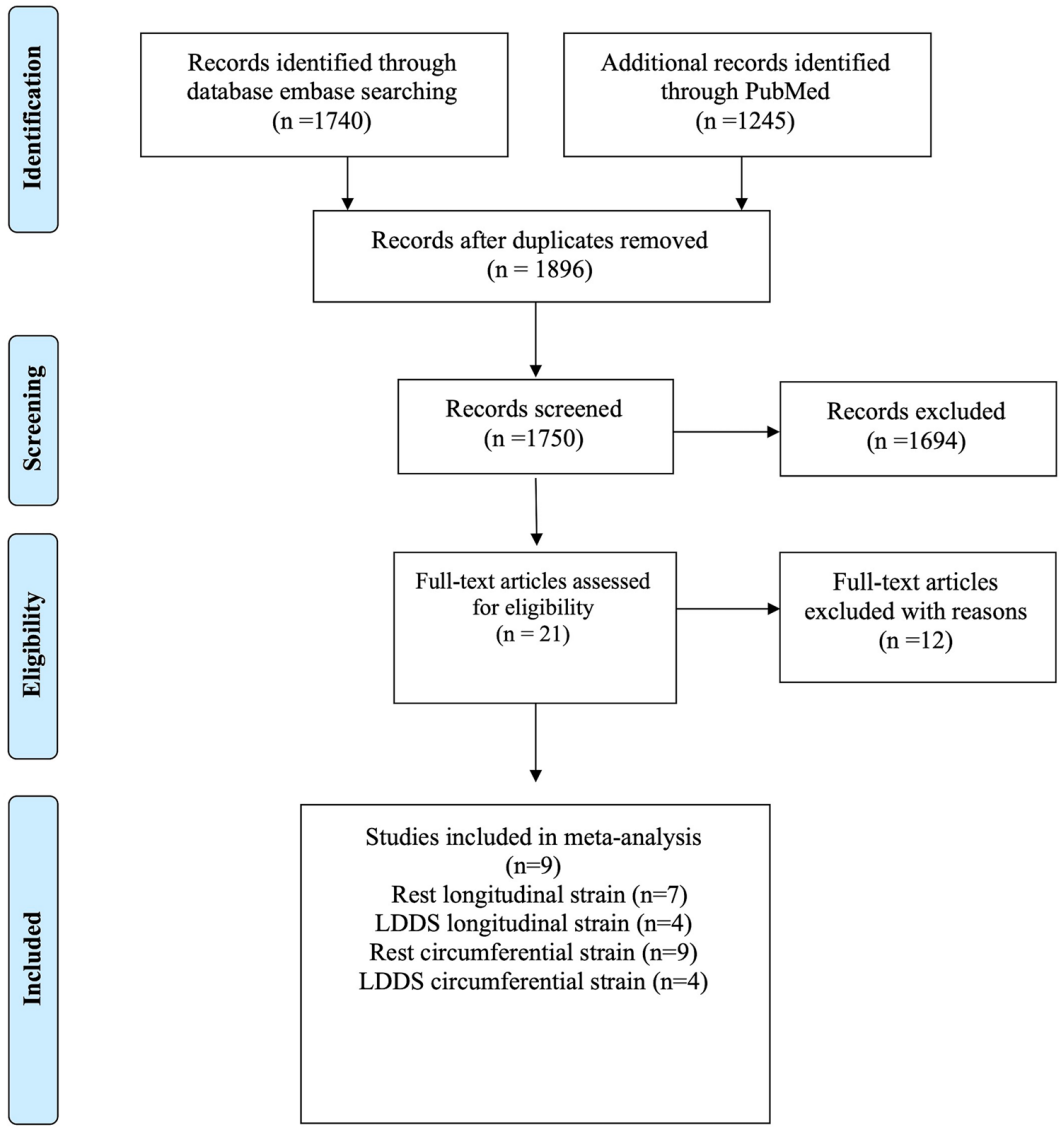
Study design. The flowchart illustrates the selection process of published reports

Using the systematic search, we identified 2985 articles, and after excluding duplicates (1089 articles), we screened 1896 studies for relevance. We excluded animal studies, non-English articles, abstracts without text, and articles unrelated to the topics (1840). Twelve articles were excluded because using other stressor than dobutamine, other reference standards, or provided no data to calculate diagnostic accuracies. Finally, nine full-text articles were found eligible for the analysis (Fig. 1).

RS was not analyzed because only one study with LDD met the inclusion criteria [12].

### Baseline characteristics

Details of studies included in the meta-analysis are summarized in Tables 1 and 2. There were five studies that included patients with chronic ischemic LV dysfunction and four studies that included patients with acute MI (two before late revascularization and two after early revascularization).

**Table 1:** Basic characters of included longitudinal strain trials

Study	Year	Patients (number)	Segments (number)	Ultrasound machine (software)	Male (%)	Age (year)	EF (%)	Revascularizati on	Myocardial ischemia (Acute/chronic)	Time interval (days)	Follow-up (months)	Functional strain (%)	Non- functional Strain (%)
*Rest LS*													
Altiok	2014	93	444	GE (EchoPAC)	89	60	56	PCI	Acute	2	6	− 13.8 ± 6.6	− 9.4 ± 6.7
Bansal	2010	55	375	GE (EchoPAC)	84	67	35	PCI/CABG	Chronic	42	9	− 11.7 ± 6.4	− 8.7 ± 7
Gong	2013	49	259	Philips (QLAB software 7.0)	74	60	42	PCI	Acute	12	1, 3, 6	− 11.6 ± 1.6	− 10.6 ± 1.3
Li	2016	33	216	Philips (QLAB software 8.1)	70	67	43	PCI	Chronic	180	1, 3, 6	− 11.5 ± 1.8	− 10.3 ± 1.4
Liu	2019	40	218	Siemens (software7.0 Siemens)	68	61	41	PCI	Acute	> 1	1, 3, 6	− 10.5 ± 5.5	− 7.5 ± 3.8
Orii	2014	35	176	GE (EchoPAC)	74	66	48	PCI	Acute	8	7	− 14 ± 5	− 10 ± 5
Wang	2016	35	219	Philips (QLAB software 8.1)	70	67	43	PCI	Chronic	180	1, 3, 6	− 12.1 ± 1.8	− 10.6 ± 1.7
*Stress LS*													
Bansal	2010	55	375	GE (EchoPAC)	84	67	35	PCI/CABG	Chronic	42	9	–14.6 ± 7.6	− 11.4 ± 7.7
Gong	2013	49	259	Philips (QLAB software 7.0)	74	60	42	PCI	Acute	12	1, 3, 6	− 17.3 ± 3.2	− 10.9 ± 2.7
Li	2016	33	216	Philips (QLAB software 8.1)	70	67	43	PCI	Chronic	180	1, 3, 6	− 16.4 ± 2.3	− 11.8 ± 1.6
Wang	2016	35	219	Philips (QLAB software 8.1)	70	67	43	PCI	Chronic	180	1, 3, 6	− 15.9 ± 3.7	− 12.6 ± 2

EF, ejection fraction during the admission; time interval, time interval between MI onset and 2DSTE analysis; Follow up, follow-up time after the revascularization

**Table 2:** Basic characters of included circumferential strain trials

Study	Year	Patients	Segments	Ultrasound machine (software)	Male (%)	Age (year)	EF (%)	Revasculariza tion	Myocardial ischemia (Acute/Chron ic)	Time interval (days)	Follow-up (months)	Functional strain (%)	Non-functional strain (%)
*Rest CS*													
Altiok	2014	93	444	GE (EchoPAC)	89	60	56	PCI	Acute	2	6	− 17.9 ± 9	− 12.7 ± 8.2
Bansal	2010	55	375	GE (EchoPAC)	84	67	35	PCI/CABG	Chronic	42	9	− 12.7 ± 9.5	− 7.8 ± 8.5
Becker	2008	53	463	GE (EchoPAC)	62	58	40	PCI/CABG	Chronic	42	9	− 14.9 ± 7	− 9.2 ± 5.3
Becker	2011	132	1001	GE (EchoPAC)	64	56	37	PCI	Chronic	42	8	− 17.8 ± 5.5	− 13.6 ± 5.4
Gong	2013	49	259	Philips (QLAB software 7.0)	74	60	42	PCI	Acute	12	1, 3, 6	− 11.6 ± 2	− 10.3 ± 1.9
Li	2016	33	216	Philips (QLAB software 8.1)	70	67	43	PCI	Chronic	180	1, 3, 6	NR	NR
Liu	2019	40	218	Siemens (software 7.0 Siemens)	68	61	41	PCI	Acute	> 1	1, 3, 6	− 9.8 ± 6	− 6.7 ± 4.2
Orii	2014	35	176	GE (EchoPAC)	74	66	48	PCI	Acute	8	7	− 20 ± 5	− 10 ± 6
Wang	2016	35	219	Philips (QLAB software 8.1)	70	67	43	PCI	Chronic	180	1, 3, 6	− 11.9 ± 2	− 10.5 ± 1.6
*Stress CS*													
Bansal	2010	55	375	GE (EchoPAC)	84	67	35	PCI/CABG	Chronic	42	9	− 13.1 ± 8.9	− 9.3 ± 7.1
Gong	2013	49	259	Philips (QLAB software 7.0)	74	60	42	PCI	Acute	12	1, 3, 6	− 14.9 ± 4	− 10.6 ± 2.9
Li	2016	33	216	Philips (QLAB software 8.1)	70	67	43	PCI	Chronic	180	1, 3, 6	− 15.8 ± 2	− 12.2 ± 1.5
Wang	2016	35	219	Philips (QLAB software 8.1)	70	67	43	PCI	Chronic	180	1, 3, 6	− 15.4 ± 4	− 12.4 ± 1.7

EF, ejection fraction during the admission; time interval, time interval between MI onset and 2DSTE analysis; Follow up, follow-up time after the revascularization

At rest, LS was evaluated in seven studies enrolling 340 patients (mean age 64 years, 75.6% men; mean EF 44%) and analyzing 1907 segments, whereas CS was evaluated in nine studies with 525 patients and 3371 segments (mean age 62.4 years, 72.7% men; mean EF 42.7%). During LDD stress, LS was evaluated in four studies with 172 patients and 1069 segments (mean age 65 years, 74.5% men; mean EF 40.8%), and CS in four studies with 172 patients and 1069 segments (mean age 65 years, 74.5% men; mean EF 40.8%). 2DSTE analysis was performed within 10 days after MI in four studies (2–12 days after MI), while five studies assessed the myocardial strain later (42–180 days after MI). The median follow-up time after the revascularization was 6 months. (Table1, 2).

### Quality assessment

**Fig. 2:**
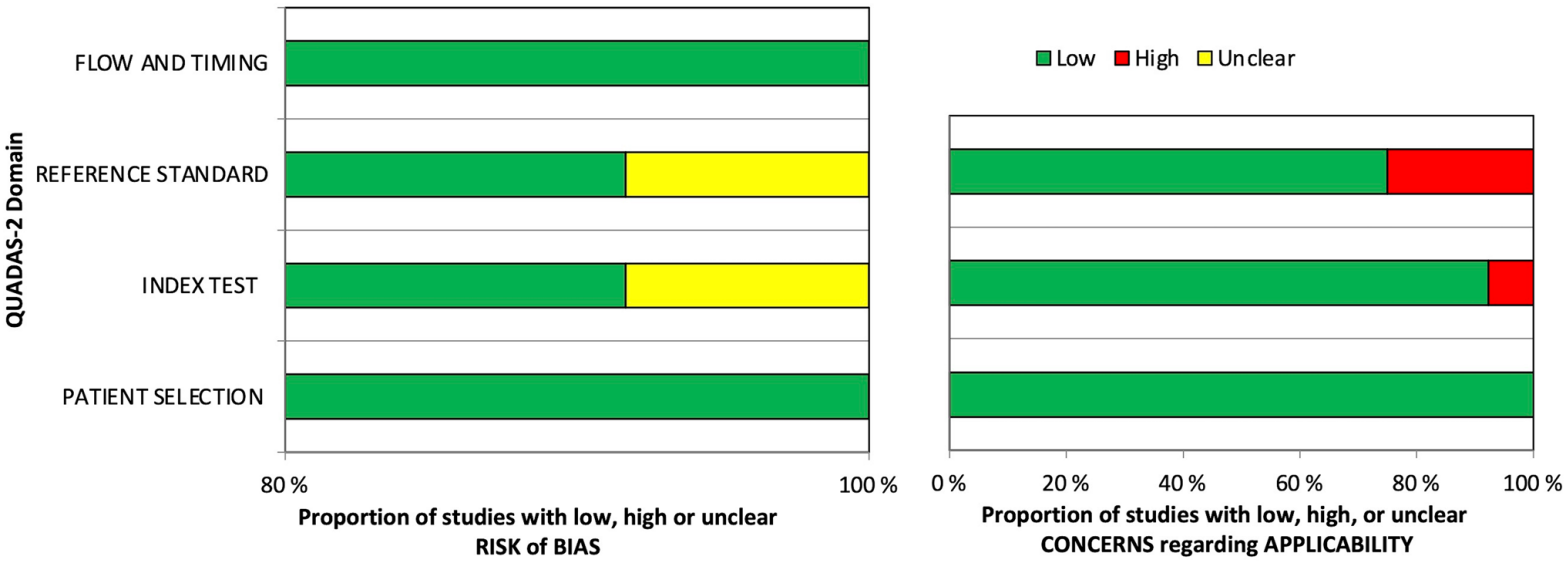
Methodological quality summary. QUADAS, quality assessment of diagnostic accuracy studies

Figure 2 shows the risk of bias and applicability concerns summary of the included studies. In one study [11], the reporting was unclear in the reference standard domain on the item (“Reference standard results blinded?”) and in the index test domain on the item (“index test results interpreted without knowledge of the results of the reference standard?”). Moreover, two studies [10, 11] were rated as high bias risk in all tests involved regarding the applicability of conduct or interpretation of the index test as well as the reference standard. Otherwise, most of the included studies showed high-quality scores and were judged acceptable (Fig. 2).

### Diagnostic accuracy

**Fig. 3:**
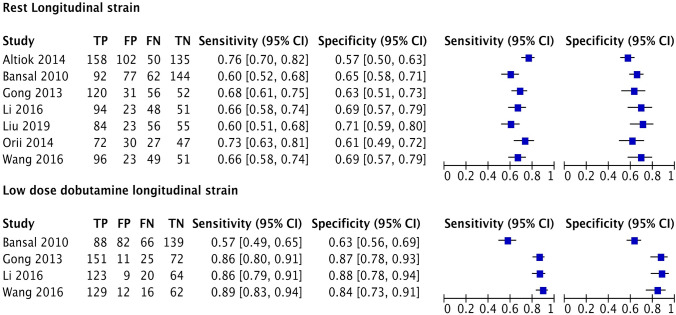
Forest Plots of Sensitivity and Specificity of included longitudinal strain trials

**Fig. 4:**
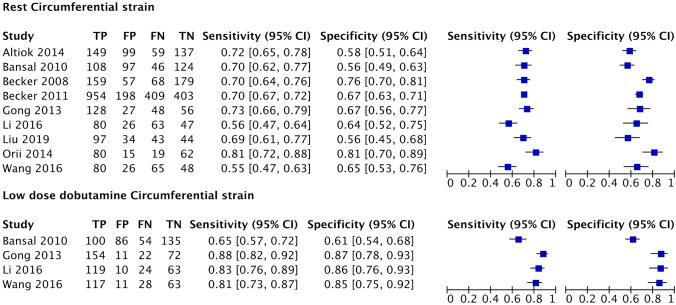
Forest Plots of Sensitivity and Specificity of included circumferential strain trials

**Fig. 5:**
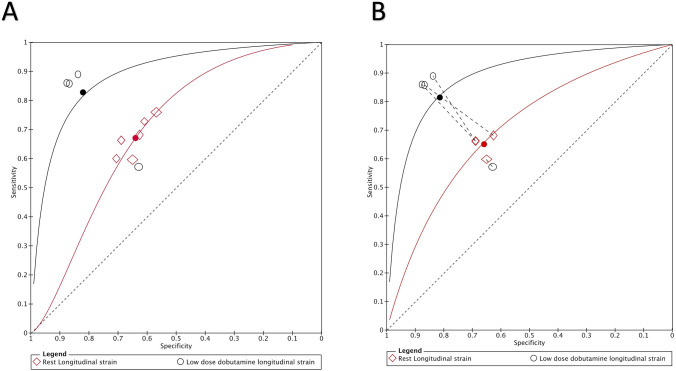
Summary ROC (SROC) curve. The SROC curves comparing the diagnostic accuracy of longitudinal strain during rest and LDD stress for predicting improvement of myocardial contractile function after revascularization. **A** Indirect comparison. **B** Direct comparison

**Fig. 6:**
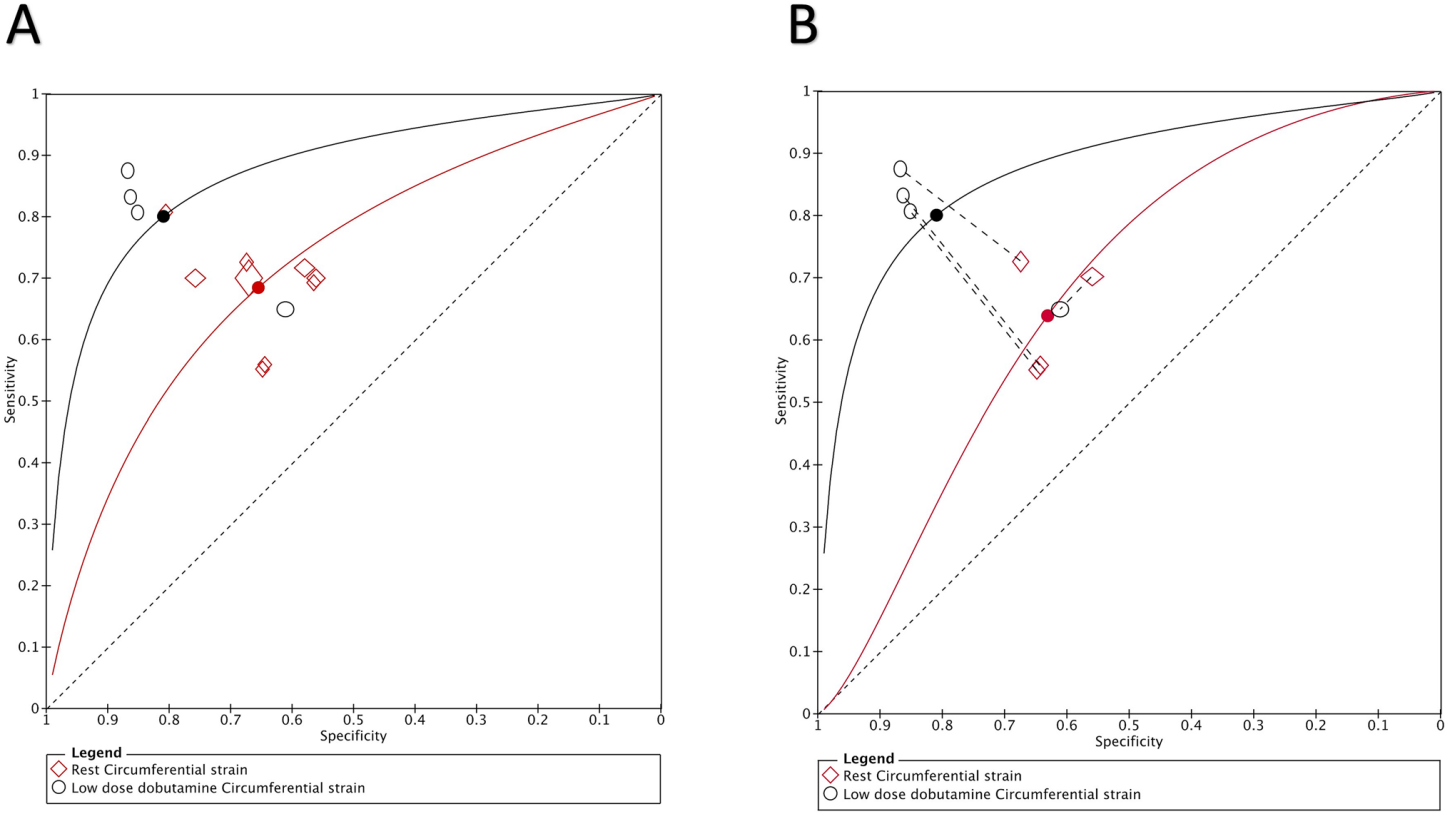
Summary ROC (SROC) curve. The SROC curves comparing the diagnostic accuracy of circumferential strain during rest and LDD stress for predicting improvement of myocardial contractile function after revascularization. **A** Indirect comparison. **B** Direct comparison

A summary of diagnostic accuracies of strain parameters in predicting regional functional recovery by bivariate models is shown in Table 3. Forest plots and ROC curves are displayed in Figs. 3, 4, 5, 6.

**Table 3:** Summary of diagnostic performance for longitudinal and circumferential strain

Longitudinal strain	RLS	Stress LS	*P* value
*Indirect comparison*			
Number of studies (segments)	7 (1907)	4 (1069)	
Sensitivity (%), (95% CI)	67.1 (62.5–71.5)	81.5 (63.7–91.7)	< 0.0001
Specificity (%), (95% CI)	64 (59.7–68.1)	81.3 (65.3–90.9)	< 0.0001
LR + (95% CI)	1.8 (1.7–2)	4.3 (1.6–12.1)	0.006
LR − (95% CI)	0.5 (0.4–0.6)	0.2 (0.1–0.6)	0.06
*Direct comparison*			
Number of studies (segments)	4 (1069)	4 (1069)	
Sensitivity (%), (95% CI)	65.1(61.2–68.8)	81.5 (63.7–91.7)	< 0.0001
Specificity (%), (95% CI)	65.9 (61.4–70.1)	81.3 (65.3–90.9)	0.005
LR + (95% CI)	1.9 (1.6–2.1)	4.3 (1.6–12.1)	0.04
LR − (95% CI)	0.5 (0.5–0.6)	0.2 (0.1–0.6)	0.1

Circumferential strain	RCS	LDDCS	*P* value
*Indirect comparison*			
Number of studies (segments)	9 (3371)	4 (1069)	
Sensitivity (%), (95% CI)	68.7 (63.9–73.1)	81.5 (66.2–90.8)	< 0.0001
Specificity (%), (95% CI)	65.7 (60–71)	81.4 (69–89.6)	0.0008
LR + (95% CI)	2 (1.7–2.4)	4.3 (1.6–11.3)	0.005
LR − (95% CI)	0.5 (0.4–0.6)	0.2 (0.1- 0.4)	0.05
*Direct comparison*			
Number of studies (segments)	4 (1069)	4 (1069)	
Sensitivity (%), (95% CI)	63.8 (54.3–72.4)	81.5 (66.2–90.8)	< 0.0001
Specificity (%), (95% CI)	61.9 (56–67.5)	81.4 (69–89.6)	< 0.0001
LR + (95% CI)	1.7 (1.5–1.9)	4.3 (1.6–11.3)	< 0.0001
LR − (95% CI)	0.5 (0.4–0.6)	0.2 (0.1–0.4)	0.005

RLS, longitudinal strain during rest, Stress LS, longitudinal strain during low dose dobutamine stress, RCS, circumferential strain during rest, LDDCS, circumferential strain during low dose dobutamine stress. LR + , positive likelihood ratio. LR − , negative likelihood ratio

Rest LS showed mean weighted sensitivity and specificity of 67.1% (95% CI: 62.5–71.5%) and 64% (95% CI: 59.7–68.1%), respectively. LR + and LR- were 1.8 (95% CI: 1.7–2) and 0.5 (95% CI: 0.4–0.6), respectively. The mean weighted LS in segments with and without functional recovery were − 12.1% (95% CI: − 12.7 to − 11.5%) vs. − 9.7% (95% CI: − 10.4 to − 9.0%), MD − 2.0% (95% CI: − 2.7 to − 1.3%), *p* < 0.001, respectively (Supplemental appendix 1: Figs. 1, 2, and 3).

LS during LDD stress was evaluated using dobutamine dose of 10 µg/kg/min in three studies and both 5 and 10 µg/kg/min in one study. The mean weighted sensitivity and specificity of LS during LDD were 81.5% (95% CI: 63.7–91.7) and 81.3% (CI: 65.3–90.9%,) respectively. The LR + was 4.3 (95% CI: 1.6–12.1) and LR- 0.2 (95% CI: 0.1–0.6). The mean weighted LS during LDD in segments with and without functional recovery were − 16.2% (95% CI: − 16.9 to − 15.4%) vs. − 11.7% (95% CI: − 12.5 to − 10.9%), MD − 4.4% (95% CI: − 5.8 to − 3.0%), *p* < 0.001, respectively (Supplemental appendix 1: Figs. 4, 5, and 6).

Rest CS showed mean weighted sensitivity of 68.7% (95% CI: 63.9–73.1%) and specificity of 65.7% (95% CI: 60–71%). LR + and LR- were 2 (95% CI: 1.7–2.4) and 0.5 (95% CI: 0.4–0.6), respectively. The mean weighted rest CS strain values in segments with and without functional recovery were − 14.7% (95% CI: − 17.1 to − 12.4) vs. − 10.2 (95% CI: − 11.7 to − 8.6), MD − 4.5% (95% CI: − 5.9 to − 3.1), *p* < 0.001, respectively (Supplemental appendix 1: Figs. 7, 8, and 9).

CS during LDD stress was evaluated using dobutamine dose of 10 µg/kg/min in three studies and both 5 and 10 µg/kg/min in one study. The mean weighted sensitivity and specificity of CS during LDD were 81.5% (CI: 66.2–90.8) and 81.4% (CI: 69–89.6%). LR + and LR- were 4.3 (95% CI: 1.6–11.3) and 0.2 (95% CI: 0.1–0.4), respectively. The mean weighted CS during LDD in segments with and without functional recovery were − 14.9% (95% CI: − 15.9 to − 13.9%) vs. − 11.2% (95% CI: − 12.2 to − 10.2%), MD − 3.7% (95% CI: − 4.3 to − 3.2), *p* < 0.001, respectively (Supplemental appendix 1: Figs. 10, 11, and 12).

Compared with rest, LS during LDD showed higher sensitivity (*p* < 0.0001) and specificity (*p* < 0.0001) for predicting functional recovery. Furthermore, the sensitivity and specificity of CS during LDD stress were superior when compared CS at rest (< 0.0001and *p* = 0.0008, respectively).

In a sub-analysis, we focused on four studies that performed a head-to-head comparison of rest and LDD strain in the same 172 patients and 1069 segments [10, 12–14]. Sensitivity and specificity of LS was higher during LDD stress than at rest (81.5%; 95% CI: 63.7–91.7 vs. 65.1%; CI: 61.2–68.8%, *p* < 0.0001, and 81.3%; CI: 65.3–90.9% vs. 65.9%; CI: 61.4–70.1%, *p* = 0.005, respectively). Similarly, these studies demonstrated the superiority of sensitivity and specificity of CS during LDD stress when compared CS at rest (81.5%; CI: 66.2–90.8 vs. 63.8%; CI: 54.3–72.4%, *p* < 0.0001, and 81.4%; CI: 69–89.6% vs. 61.9%; CI: 56–67.5%, *p* < 0.0001, respectively).

Considering the substantial degree of heterogeneity observed in the summary estimates, we performed a further sensitivity analysis by excluding the outlier studies. After exclusion of three studies evaluating CS at rest [13, 14, 20] and one study evaluating LS and CS during LDD stress [10], the diagnostic accuracy of CS at rest as well as CS and LS during LDD stress were slightly improved. (Supplemental appendix 2: Table 1).

### Assessment of heterogenicity

Visual inspection of the forest plot for rest CS sensitivity revealed three potential outliers. [13, 14] reported rest CS sensitivities lower than the other studies. The most likely explanation for this is that the evaluation of functional recovery was performed already at 1 month after PCI in 51% of patients, whereas the duration of follow-up was longer (3–9 months) in other studies. The short time may not be enough for the recovery of cardiac function after the revascularization. Another possible outlier for rest CS sensitivity was the high estimate in [20], which may be explained by the small size of the study population. A small sample size produces an imprecise estimate of the diagnostic accuracy and may affect the reliability of meta-analyses [21, 22]. A possible outlier in sensitivity and specificity estimates of LS and CS during LDD stress was the low estimate in [10]. Regarding CS, this may be associated with incorporation of only mid-ventricular short axis images in the analysis. Apical segments could be particularly important for the assessment of myocardium supplied by the anterior coronary circulation. Besides, strain results will differ depending on which segment will be excluded, as apical segments usually show higher strain values than other short-axis views [23].

### Meta-regression

We performed multivariable meta-regression to further evaluate factors that impact diagnostic accuracy of LS and CS (Table 4). In these analyses, LDD remained strongly associated with high sensitivity and specificity. Other predictors included preserved EF, which was associated with sensitivity of LS and specificity of CS. Furthermore, analysis of viability shortly after an MI was associated with high sensitivity and specificity, whereas male sex was associated with low specificity of CS. Other covariates did not significantly influence the diagnostic performance of LS and CS.

**Table 4:** Multivariate meta-regression for potential variables affecting diagnostic performance

	Beta coefficient	Sensitivity		Beta coefficient	Specificity	
		SE	*P*		SE	*P*
*Longitudinal strain*						
Age	− 0.006	0.014	0.71	− 0.003	1.017	0.851
Male	− 0.002	0.018	0.211	− 0.027	0.016	0.095
EF	0.063	0.011	0.003	− 0.006	0.019	0.773
Follow up	− 0.133	0.096	0.748	− 0.093	0.09	0.300
Time interval	0.002	0.002	0.667	0.001	0.002	0.694
LDD stress	0.87	0.185	< 0.001	0.585	0.198	0.004
*Circumferential strain*						
Age	0.035	0.034	0.30	0.079	0.026	0.002
Male	− 0.025	0.012	0.05	− 0.071	0.013	< 0.001
EF	0.035	0.02	0.06	0.05	0.013	< 0.001
Follow up	− 0.044	0.06	0.42	0.004	0.044	0.93
Time interval	− 0.004	0.002	0.005	− 0.003	0.001	0.021
LDD stress	0.81	0.181	< 0.001	0.643	0.148	< 0.001

SE, Standard error; Follow up, follow-up time after the revascularization; Time interval, time interval between MI onset and 2DSTE analysis; EF, ejection fraction during the admission. All variables were continuous except LDD stress (Factor)

## Discussion

This is a first meta-analysis that systematically evaluated the performance of 2DSTE for predicting improvement of segmental LV contractile function after revascularization and explored the factors that may influence its performance in patients with chronic ischemic LV dysfunction or recovering from acute MI. Overall, we found that in both clinical conditions LS and CS during LDD stress provided equally high sensitivity and specificity for identifying reversible myocardial dysfunction, whereas LS and CS at rest showed lower accuracy. Based on these results, LS and CS during LDD stress rather than at rest should be used for the assessment of myocardial viability.

Detection of myocardial viability is of particular importance for revascularization decisions in patients with chronic ischemic LV dysfunction [3, 5]. However, recovery of myocardial function after acute MI is another setting in which accuracy of viability tests can be evaluated. Meta-regression analysis indicated that accuracy of viability assessment did not differ between these settings, except for potentially improved sensitivity of CS in the setting of an acute MI. Therefore, these settings were combined in order to increase the sample size of the analysis.

Evaluation of myocardial viability with LDD echocardiography has been mainly performed by subjective visual assessment of wall-thickening responses, which analysis is highly operator-dependent. It requires experience and visualization of endocardial border may be challenging [18]. Strain parameters assessed by tissue Doppler imaging (TDI) during DSE have been shown to be feasible, and their use in combination with visual wall motion analysis during DSE may facilitate the evaluation of viability [24]. However, the clinical application of myocardial strain evaluation with TDI has been limited due to its susceptibility to signal noise, requirement for acquisition of specific images, and dependency of angle between ultrasound beam and deformation [25]. 2DSTE is independent of ultrasound angle, can measure myocardial deformation in many directions and can be evaluated from standard grey scale images. It has been shown to be potentially useful for the detection of ischemic wall motion abnormalities at rest and during dobutamine stress with low inter- and intra-observer variability [26–28]. Furthermore, previous studies have shown that global longitudinal strain by 2DSTE is related to infarct size and may serve as a useful tool to predict recovery of LV function after acute MI at 1-year follow-up [29]. In addition, 2DSTE parameters were superior to LVEF and WMA in the risk stratification for long-term outcome [30].

Some studies have indicated that 2DSTE has a comparable diagnostic performance with SPECT [12] and contrast-enhanced MRI [31] to predict segmental functional recovery. However, other studies have indicated that the accuracy of 2DSTE is limited and inferior compared with contrast-enhanced MRI [11] and TDI [10].

2DSTE is a relatively novel imaging technique and it has not been included in previous meta-analyses on the detection of myocardial viability [1]. The main finding of our study is that 2DSTE performed in combination with LDD stress has superior diagnostic accuracy for recognizing reversible myocardial dysfunction as compared to rest 2DSTE alone. The improved accuracy of 2DSTE with LDD stress can be attributed to the fact that dobutamine is an adrenoreceptor agonist, and a low dose can enhance systolic myocardial function by improving coronary blood flow. These findings indicate that LDD stress should be preferred over rest when evaluating viability by 2DSTE. In addition to these, radial strain during LDD stress has also been proposed as a marker of viability in one study [12], but this remains to be confirmed in further studies.

Previous published data demonstrated that LS and CS during stress echocardiography resulted in similar accuracy levels for quantification of myocardial function [32]. Our results on the same line which showed that LS and CS during LDD were almost equally superior to Rest LS and CS with higher sensitivities and specificities. However, from a practical clinical perspective, CS is often more difficult to perform than LS due to the increase variability and lack of reproducibility [33]. Therefore, LS could be more feasible than CS in the clinical sitting even both showed comparable diagnostic accuracies.

Previous studies found global longitudinal strain of 11.3% or 13.7% as the best cut-off value for predicting improvement of global left ventricle function (≥ 5% increase in ejection fraction) after acute myocardial infarction [34]. Regarding regional longitudinal strain, studies have found variable cut-off values from 4.5% in chronic ischemic cardiomyopathy to 13.5% after acute myocardial infarction to identify non-viable myocardium based on gadolinium late enhancement [35, 36]. In our meta-analysis it was not possible to define exact cut-off values for regional strain to predict functional improvement, but average values in viable and non-viable regions in different studies are shown in Tables 1 and 2.

## Clinical implications

The availability of an accurate, non-invasive method for defining the viable myocardium after MI is an important factor in the clinical decision making and prognostic evaluation. Assessment of contractile reserve by 2DSTE is a novel echocardiographic metric that could be readily integrated into the current clinical setting as an adjunct to visual assessment [37]. Our meta-analysis shows that the diagnostic accuracies of 2DSTE during LDD stress are similar to those reported for visual wall-motion analysis during LDD stress published by Schinkel et al. (sensitivity 80% and specificity 78%) [1].

## Study limitations

The main limitation of this meta-analysis is relatively small number of included studies. Including only studies that evaluated the accuracy of 2DSTE for the prediction of regional functional recovery as the gold standard contributed to this. The definition of viability in the included studies was improvement of wall motion after revascularization that is qualitative and subjective. Moreover, studies using other inotropic agents were excluded from this meta-analysis because of few studies. The limited number of studies, in turn, decreased our ability to perform subgroup analyses and to evaluate some possible causes of heterogenicity, such as acute MI vs. chronic LV dysfunction. Furthermore, we could not compare strain analysis with visual wall motion analysis in the detection of viability, because this was performed in only one study [14].

Our meta-analysis showed a considerable heterogeneity of sensitivity and specificity between studies. This observed heterogeneity is likely to arise through variety in methodological characteristics between different studies and the basic differences among the patients in the included studies. However, in meta-regression analysis of the current study, the methodological characteristics and basic differences did not possess any significant heterogeneity. Furthermore, due to the absence of information in the included studies, we could not evaluate the impact of heart rate and systolic blood pressure on the diagnostic accuracy of LS and CS during LDD. However, LDD is associated with only a mild hemodynamic response [38].

This meta-analysis included studies with different vendors. A recent study showed no significant difference in identifying regional dysfunction and myocardial scar [39]. However, the variability of segmental measurements between different vendors was high for clinical use. Thus, vendor variability could affect the results presented.

Finally, in contrast to global strain, segmental strain measurements still have higher variability. Thus, segmental strain measurements should be used for clinical decision-making, monitoring, and research with caution [40].

## Conclusions

Longitudinal and circumferential strain analysis combined with low dose dobutamine stress accurately detects myocardial viability as defined by recovery of wall motion abnormality during follow-up. Compared with strain analysis at rest, the use of dobutamine stress significantly improved the diagnostic performance and therefore it can be recommended to improve the accuracy of the 2DSTE for detection of reversible myocardial dysfunction.

## Supplementary Information

Below is the link to the electronic supplementary material.

Supplementary file1 (PDF 1078 KB)

Supplementary file2 (DOCX 18 KB)
