# Supplementary material for: Speckle-tracking echocardiography for predicting improvement of myocardial contractile function after revascularization: a meta-analysis of prospective trials
Source: Int J Cardiovasc Imaging. 2022 Nov 11;39(3):541–53. doi: 10.1007/s10554-022-02753-2 (PMC9947084; doi:10.1007/s10554-022-02753-2)
Supplement: Supplementary file 1 — Supplementary file1 (PDF 1078 KB) [file 10554_2022_2753_MOESM1_ESM.pdf]

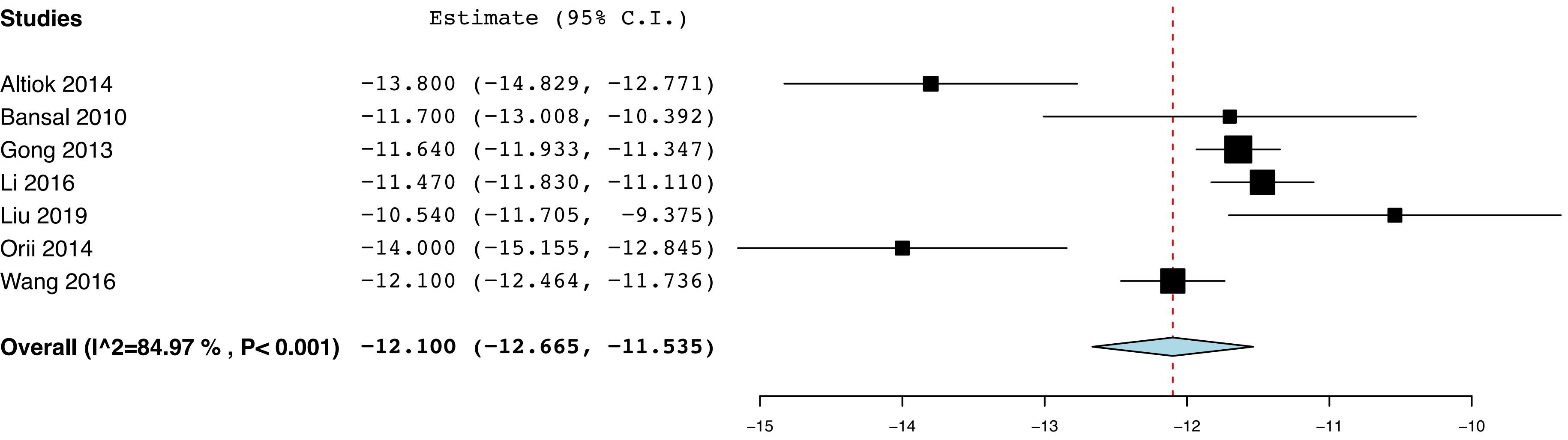

Figure 1.The mean weighted rest longitudinal strain values in segments with functional recovery

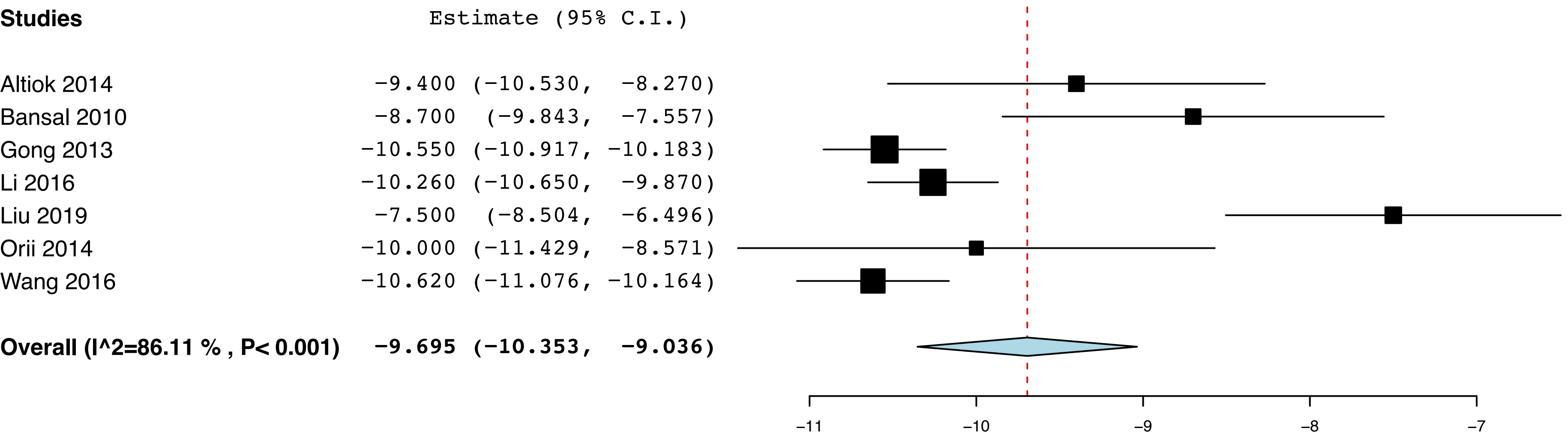

Figure 2.The mean weighted rest longitudinal strain values in segments without functional recovery

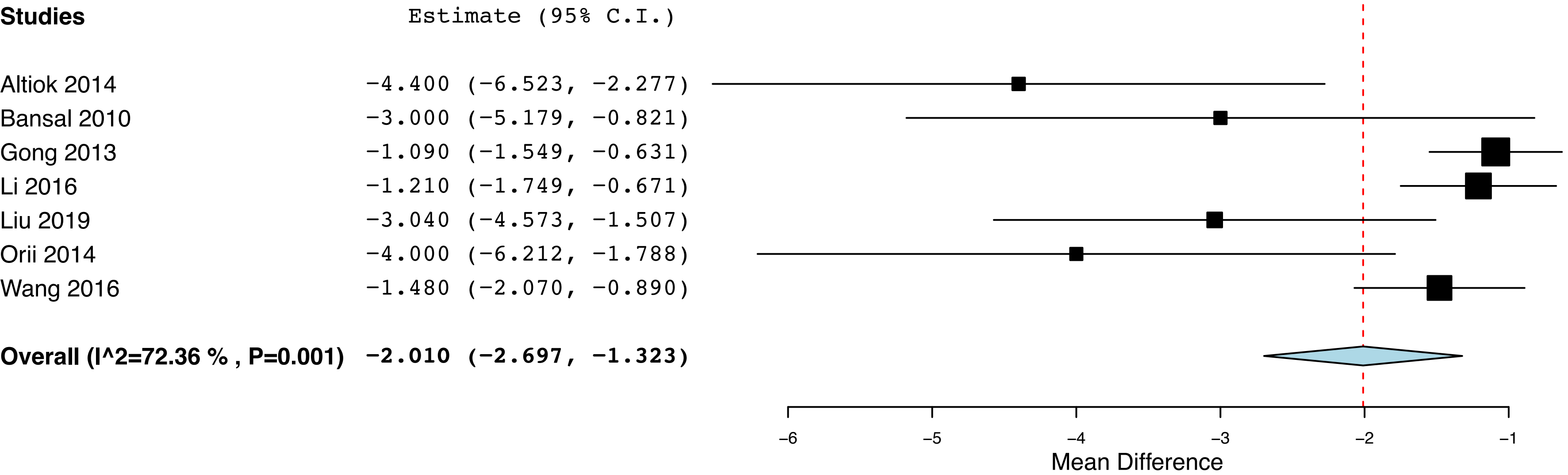

Figure 3.The mean difference in rest longitudinal strain values in segments with and without functional recovery

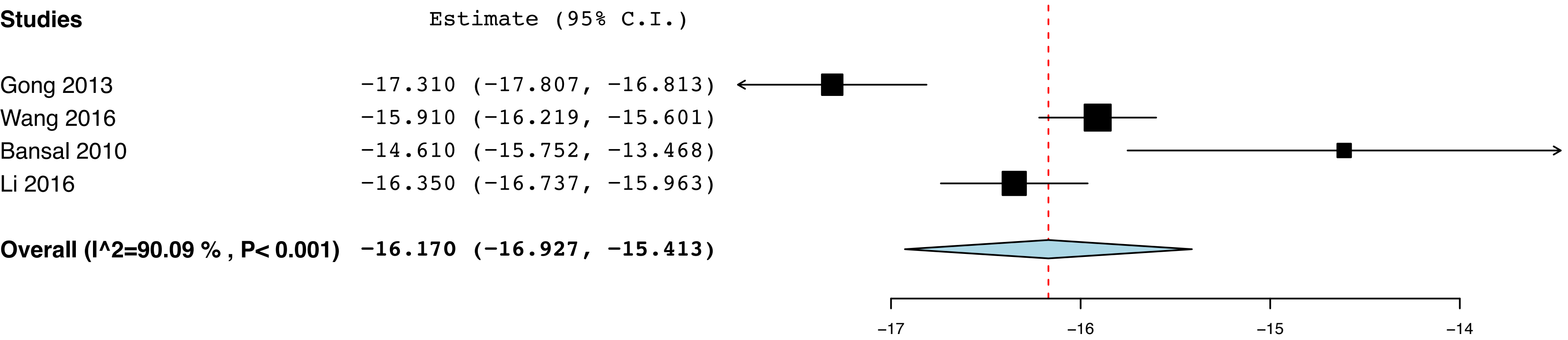

Figure 4.The mean weighted LDDS longitudinal strain values in segments with functional recovery

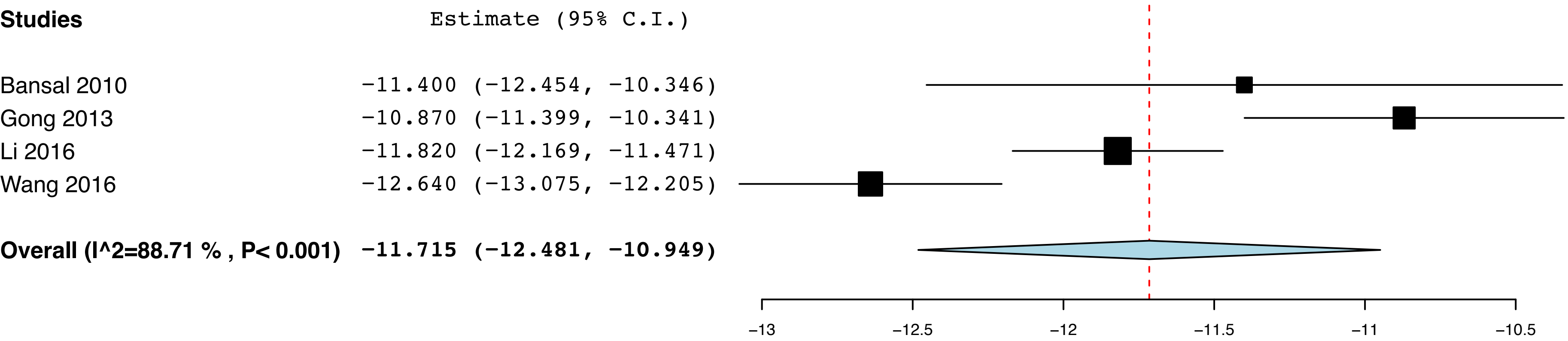

Figure 5.The mean weighted LDDS longitudinal strain values in segments without functional recovery

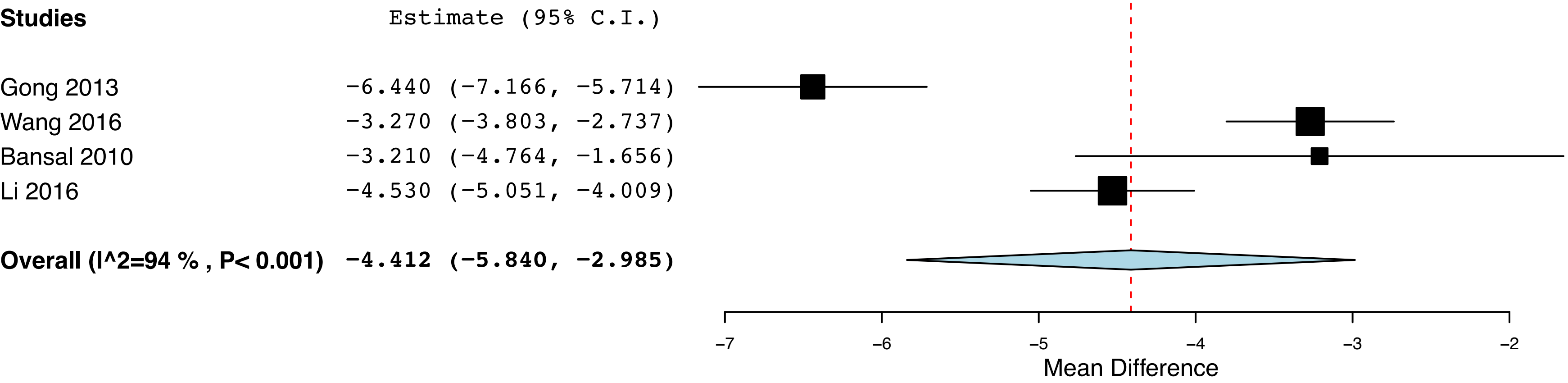

Figure 6.The mean difference in LDDS longitudinal strain values in segments with and without functional recovery

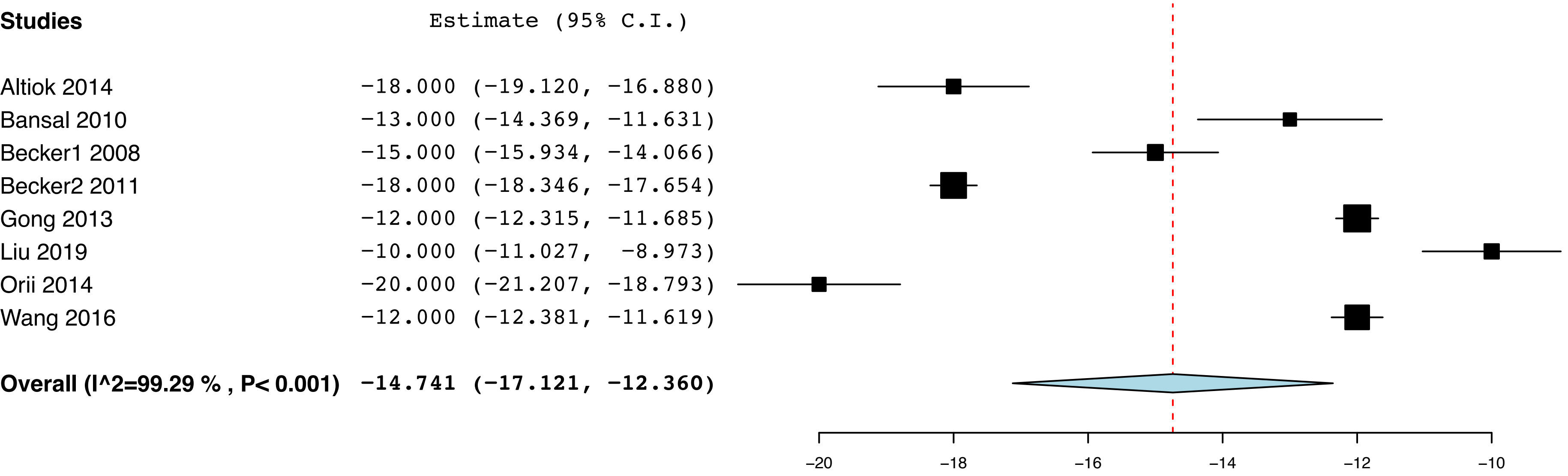

Figure 7.The mean weighted rest circumferential strain values in segments with functional recovery

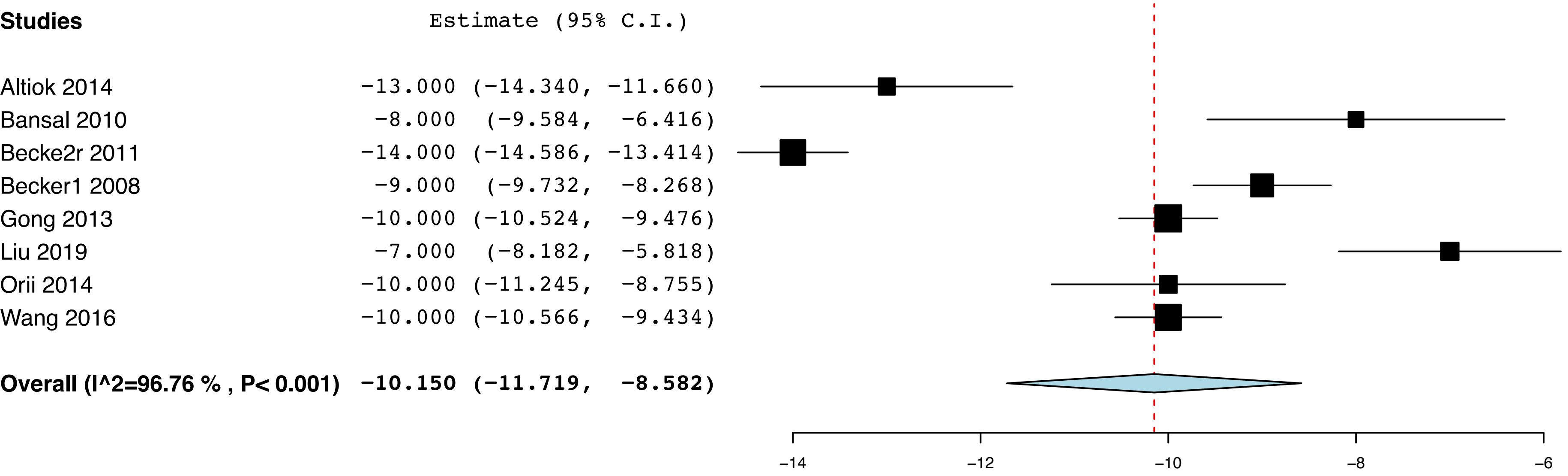

Figure 8.The mean weighted rest circumferential strain values in segments without functional recovery

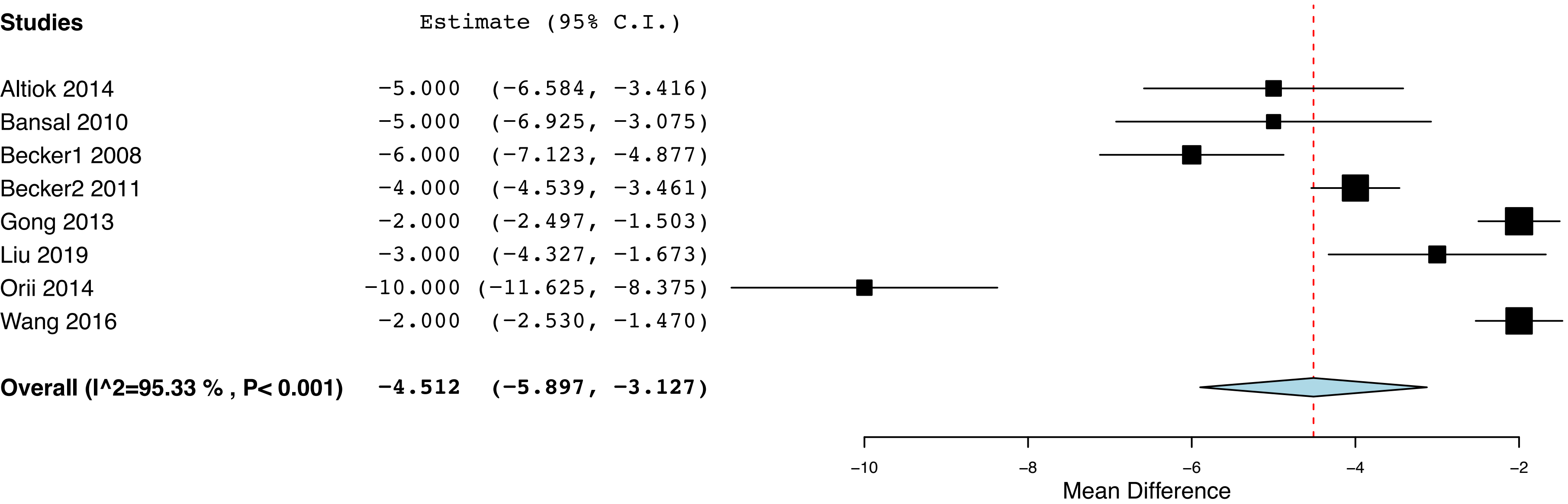

Figure 9.The mean difference in rest circumferential strain values in segments with and without functional recovery

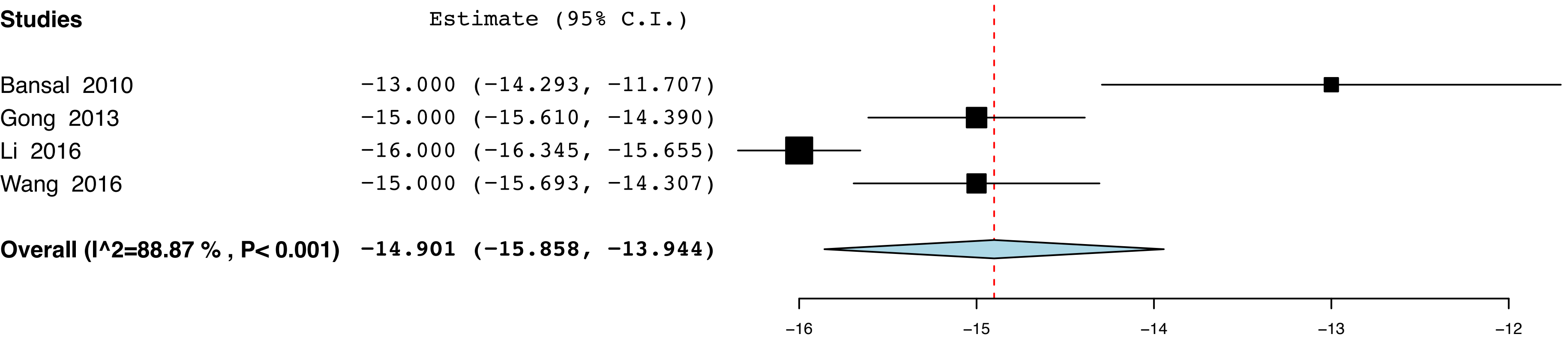

Figure 10.The mean weighted LDDS circumferential strain values in segments with functional recovery

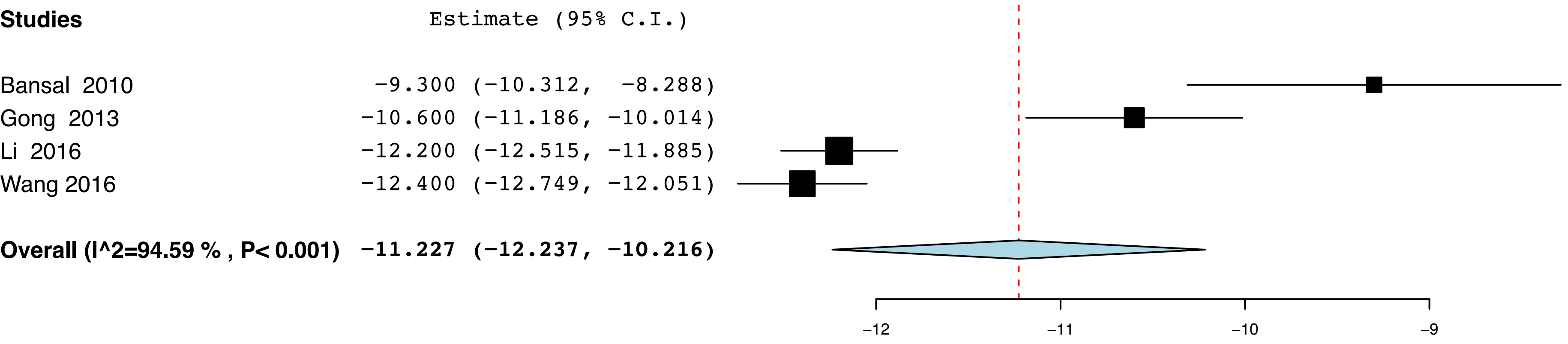

Figure 11.The mean weighted LDDS circumferential strain values in segments without functional recovery

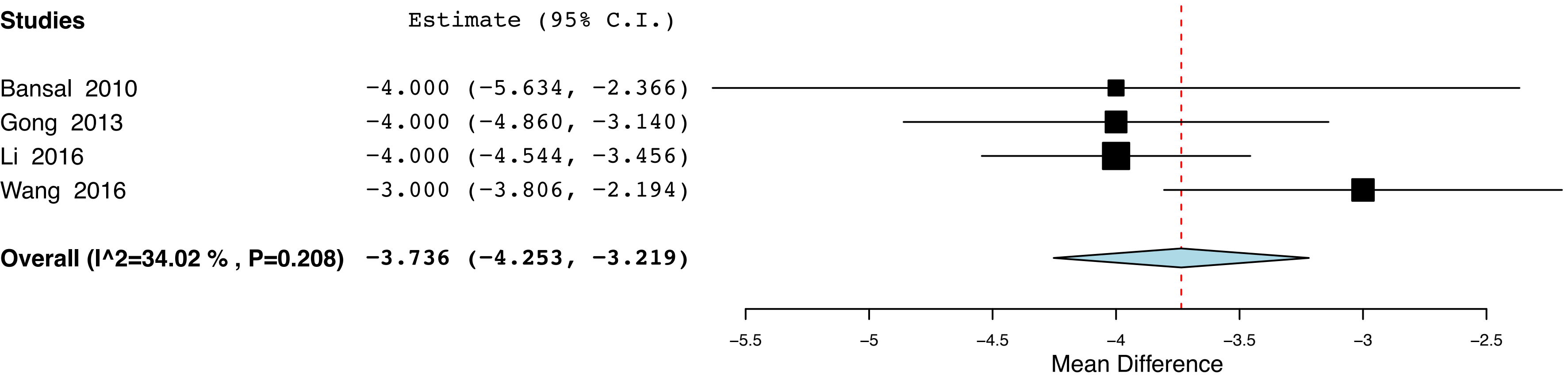

Figure 12.The mean difference in LDDS circumferential strain values in segments with and without functional recovery
