# Supplementary material for: Speckle-tracking echocardiography for predicting improvement of myocardial contractile function after revascularization: a meta-analysis of prospective trials
Source: Int J Cardiovasc Imaging. 2022 Nov 11;39(3):541–53. doi: 10.1007/s10554-022-02753-2 (PMC9947084; doi:10.1007/s10554-022-02753-2)
Supplement: Supplementary file 2 — Supplementary file2 (DOCX 18 KB) [file 10554_2022_2753_MOESM2_ESM.docx]

**SUPPLEMENTAL APPENDIX 2**

Table 1. Summary of diagnostic performance for longitudinal and circumferential strain before and after outlier exclusion.

| Longitudinal strain |  |  |  |
| --- | --- | --- | --- |
|  | **RLS** | **Stress LS** | ***P* value** |
| Before outlier exclusion |  |  |  |
| Number of studies (segments) | 7 (1907) | 4 (1069) |  |
| Sensitivity (%), (95% CI) | 67.1 (62.5 - 71.5) | 81.5 (63.7 - 91.7) | <0.0001 |
| Specificity (%), (95% CI) | 64 (59.7 - 68.1) | 81.3 (65.3 - 90.9) | <0.0001 |
| LR+ (95% CI) | 1.8 (1.7 - 2) | 4.3 (1.6 - 12.1) | 0.006 |
| LR- (95% CI) | 0.5 (0.4 - 0.6) | 0.2 (0.1 - 0.6) | 0.06 |
|  |  |  |  |
| After outlier exclusion |  |  |  |
| Number of studies (segments) | 7 (1907) | 3 (694) |  |
| Sensitivity (%), (95% CI) | 67.1 (62.5 - 71.5) | 86.8 ( 83.4- 89.6 ) | <0.0001 |
| Specificity (%), (95% CI) | 64 (59.7 - 68.1) | 86 (65.3 - 90.9) | <0.0001 |
| LR+ (95% CI) | 1.8 (1.7 - 2) | 6.2 (4.5 - 8.6) | <0.0001 |
| LR- (95% CI) | 0.5 (0.4 - 0.6) | 0.2 (0.1 - 0.6) | 0.06 |
|  |  |  |  |
| Circumferential strain |  |  |  |
|  | **RCS** | **LDDCS** | ***P* value** |
| Before outlier exclusion |  |  |  |
| Number of studies (segments) | 9 (3371) | 4 (1069) |  |
| Sensitivity (%), (95% CI) | 68.7 (63.9 - 73.1) | 81.5 (66.2 - 90.8) | <0.0001 |
| Specificity (%), (95% CI) | 65.7 (60 - 71) | 81.4 (69 - 89.6) | 0.0008 |
| LR+ (95% CI) | 2 (1.7 - 2.4) | 4.3 (1.6 - 11.3) | 0.005 |
| LR- (95% CI) | 0.5 (0.4 - 0.6) | 0.2 (0.1- 0.4) | 0.05 |
|  |  |  |  |
| After outlier exclusion |  |  |  |
| Number of studies (segments) | 6 (2760) | 3 (694) |  |
| Sensitivity (%), (95% CI) | 70.3 ( 68.4 - 72.1) | 83.9 ( 79.5 - 87.5 ) | <0.0001 |
| Specificity (%), (95% CI) | 64 ( 57.7 - 69.9) | 86.1 (81 - 90) | <0.0001 |
| LR+ (95% CI) | 1.9 (1.6 - 2.3) | 6 (4.3 - 8.3) | 0.03 |
| LR- (95% CI) | 0.5 ( 0.4 - 0.5) | 0.2 (0.1 - 0.5) | 0.05 |

RLS, longitudinal strain during rest, Stress LS, longitudinal strain during low dose dobutamine stress, RCS, circumferential strain during rest, Stress CS, circumferential strain during low dose dobutamine stress. LR+, positive likelihood ratio. LR-, negative likelihood ratio.
